# *DYNC1H1* variant associated with epilepsy: Expanding the phenotypic spectrum

**DOI:** 10.1016/j.ebr.2022.100580

**Published:** 2022-12-28

**Authors:** Chi-Ting Chung, Ni-Chung Lee, Sung-Pin Fan, Miao-Zi Hung, Yen-Heng Lin, Chih-Hao Chen, Tun Jao

**Affiliations:** a Department of Neurology, National Taiwan University Hospital, Taipei, Taiwan; b Department of Neurology, National Taiwan University, Taipei, Taiwan; c Department of Pediatrics and Medical Genetics, National Taiwan University Hospital, Taipei, Taiwan; d Department of Medical Genetics, National Taiwan University, Taipei, Taiwan; e Department of Medical Imaging, National Taiwan University, Taipei, Taiwan

**Keywords:** CMT, Charcot–Marie–Tooth disease, EEG, electroencephalography, ID, intellectual disability, MCD, malformation of cortical development, MRI, magnetic resonance imaging, SMALED, spinal muscular atrophy with lower extremity-predominance, Epilepsy, Intellectual disability, Malformations of cortical development, Neurodevelopmental delay, Pathogenic *DYNC1H1* variant

## Abstract

*DYNC1H1* variants are associated with peripheral neuronal dysfunction and brain morphology abnormalities resulting in neurodevelopmental delay. However, few studies have focused on the association between *DYNC1H1* variants and epilepsy. Herein, we report a case of drug-resistant focal epilepsy associated with a pathogenic variant of *DYNC1H1*. We further summarized the clinical, genetic, and neuroimaging characteristics of patients with *DYNC1H1* variant–associated epilepsy from the relevant literature. This report expands the phenotypic spectrum of *DYNC1H1*-related disorder to include early-onset epilepsy, which is frequently associated with neurodevelopmental delay and intellectual disability, malformations of cortical development, and neuromuscular, ophthalmic, and orthopedic involvement.

## Highlights

• *DYNC1H1* is a key factor for developmental and epileptic encephalopathies.
• Besides developmental and neuromuscular problems, epilepsy may develop in the *DYNC1H1* variant.
• Brain malformation is common in *DYNC1H1* variant-associated epilepsy.
• *DYNC1H1* variant-associated epilepsy develop in the motor domain by *de novo* mutations.
• Early-onset epilepsy may be an extended phenotype of *DYNC1H1*-related disorders.

## Introduction

The cytoplasmic dynein 1 heavy chain 1 gene (*DYNC1H1*; OMIM #600112) is crucial for intracellular motility because it encodes DYNC1H1—the largest subunit of the dynein complex—and facilitates the binding of dynein complexes to microtubules [1]. Heterozygous variants of this gene are associated with abnormal brain morphologies and motor and sensory neuronal defects [2], including axonal Charcot–Marie–Tooth disease (CMT) type 20 (OMIM #614228), spinal muscular atrophy with lower extremity predominance (SMALED)1 (OMIM #158600) [3], and malformations of cortical development (MCDs) and mental retardation 13 (OMIM #614563) [4]. Recently, because of the increasing number of overlapping phenotypes, *DYNC1H1-*related disorders have been reclassified as follows: *DYNC1H1*-related central nervous system disorders, *DYNC1H1*-related neuromuscular disorders, *DYNC1H1*-related combined disorders by Amabile et al. [5], or including other classifications as *DYNC1H1*-neuromuscular disorders (NMDs) and *DYNC1H1*-neurodevelopmental disorders by Becker et al. [6].

Although *DYNC1H1* variants may be associated with brain malformations, which implies possible epileptogenesis, the association between *DYNC1H1* variants and epilepsy remains unclear. Here, we report the case of a patient with refractory epilepsy associated with a pathogenic *DYNC1H1* variant who presented with intellectual disability (ID), dysmorphism, and brain malformation. In addition, we summarize the clinical, genetic, and neuroimaging characteristics of patients with *DYNC1H1* variant–associated epilepsy from the relevant literature.

## 2 Case presentation

A 23-year-old woman was born term to nonconsanguineous healthy parents. The patient had a normal birth and had no family history of seizures or neurological disorders. At the age of 11 months, she had experienced episodes of left-side focal to bilateral tonic-clonic seizures with cyanosis after a 2-week course of fever with upper airway symptoms. Electroencephalography (EEG) revealed considerable focal (right side) slow wave activity. Head computed tomography revealed bilateral widening of the sylvian fissure. Although aseptic encephalitis was initially suspected, no laboratory evidence supported this impression. The patient received antiseizure medications, which were discontinued after discharge. No convulsion was noted in the following years. Her medical history revealed a global developmental delay at the age of 11 months, which corresponded to the motor developmental skills of an 8-month-old infant. ID was noted during a follow-up assessment, and the patient attended a special education school. She required assistance with activities of daily living and could not work. After reaching adolescence, she underwent corrective surgeries for bilateral cataracts.

The patient experienced drug-resistant epilepsy at the age of 16 years. It started with a startle response, followed by staring, oral automatisms, and semipurposeful limb movements, such as tearing clothes, kicking, and dashing out suddenly. The episode lasted for approximately 2 min. She received > 5 types of antiseizure medications, namely levetiracetam (2,000 mg/day), topiramate (200 mg/day), lacosamide (100 mg/day), valproic acid (500 mg/day), and clonazepam (1 mg/day). However, she continued to experience approximately-three seizures per week, which were more severe during menstruation and stress.

Physical findings at presentation were as follows: long face, epicanthus, telecanthus, and prominent supraorbital ridges. Neurological examinations revealed an alert but uncooperative attitude, irrelevant speech, and childish behaviors; she had full muscle power, no specific sensory complaints, intact coordination, and no outstanding extrapyramidal signs. Video EEG indicated focal impaired awareness motor seizures. The episodes began with the abrupt onset of oral automatism involving chewing movements, which then turned toward her left side and involved the tonic movement of her left arm and the tonic posturing of all four limbs. Clinical seizures were correlated with the findings on video EEG, which showed trains of sharply contoured sharp waves and spike-and-wave complexes at 5–6 Hz and 30–80 µV, lasting for approximately 150–160 s with an emphasis on the right frontotemporal areas and occasional bilateral synchronization and generalization. Before an episode, occasionally scattered focal blunt or sharply contoured waves were noted at 4–7 Hz and 20–50 µV in the right hemisphere with phase reversal at F8.

**Fig. 1:**
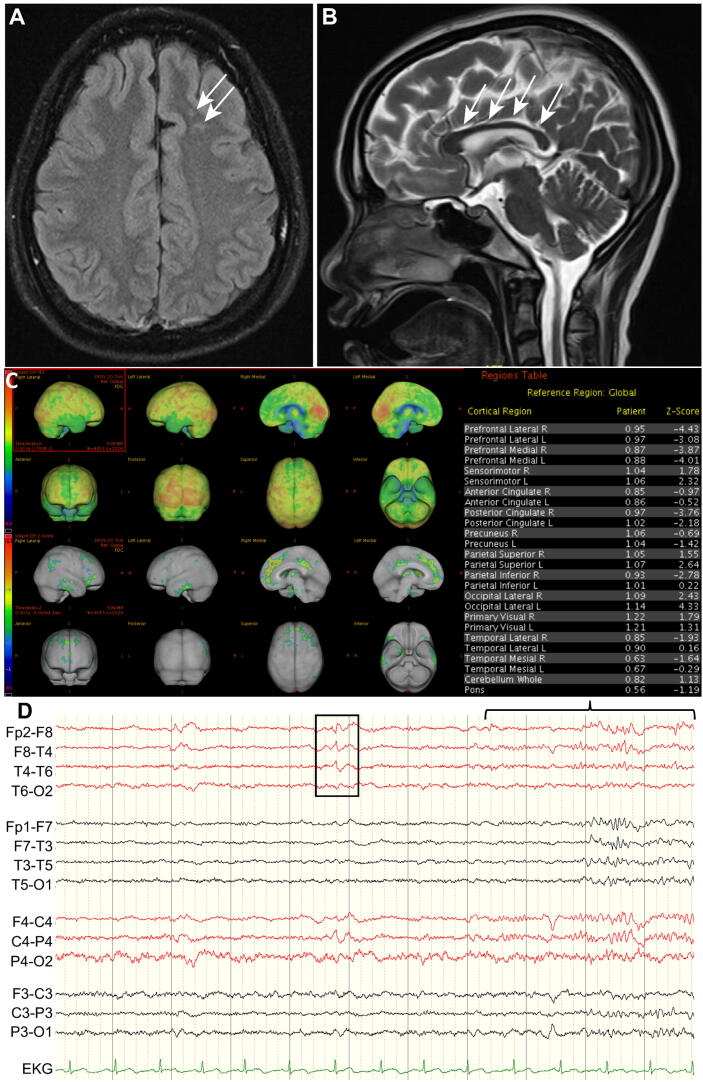
Neuroimages and video-electroencephalographic findings of the patient. (A) Axial view of a scan obtained through brain magnetic resonance imaging (MRI) fluid-attenuated inversion recovery showing several nodules at the left frontal centrum semiovale with a signal intensity similar to that of the gray matter (arrows); the largest one was approximately 10 mm in diameter. (B) MRI coronal view revealing a relatively thin corpus callosum (arrows). (C) Three-dimensional demonstration of brain ^18^F-fluorodeoxyglucose positron emission tomography image with quantification parameters indicating reduced metabolism in the bilateral frontal lobes, right parietotemporal lobes, and the bilateral posterior cingulate but with more reduction on the right side. (D) Video-electroencephalography showing focal sharp waves and spike-and-wave complexes with phase reversals at F8 (square frame), followed by fast activities originating from the right hemisphere after a few seconds (bracket).

The patient’s laboratory results, including routine biochemistry, electrolytes, and autoimmune profile, were normal. Brain magnetic resonance imaging (MRI) scan revealed a thin corpus callosum and several nodular heterotopias in the left centrum semiovale. Interictal brain ^18^F-fluorodeoxyglucose positron emission tomography indicated reduced metabolism in the right parietotemporal lobes, bilateral frontal lobes, and posterior cingulate (Fig. 1). Ophthalmological fundus examination and retinal photography suggested no degeneration of the retina or optic nerves. Cardiac echo results revealed no intracardiac shunt or septal defect.

Eventually, exome sequencing was performed; the results revealed a heterozygous missense variant NM_001376.5:c.10348G > A (p.E3450K) in exon 20 of *DYNC1H1* (Supplementary Table). In silico analysis, this variant is considered to be responsible for damaging the structure and function of the protein. It is rarely found in the general population; only one case is reported in ClinVar (variation ID: 453004). The variant was regarded as pathogenic according to the interpretation criteria outlined by the American College of Medical Genetics and Genomics. Single-nucleotide polymorphism arrays revealed no pathogenic copy number variants. The patient was presumed to have had *de novo* mutations in *DYNC1H1* because although the genetic data of her family members were unavailable, they appeared healthy without epilepsy or any other neurological disorders.

## 3 Discussion

*DYNC1H1* consists of several major domains, including the stem domain (amino acid [aa] residues 1–1,867), motor domain (aa residues 1,868–3,168 and 3,553–4,221), and a stalk/microtubule-binding domain (stalk/ MTBD; aa residues 3,189–3,500). A motor domain comprises a ring of siX aAA + domains, which help localize ATP bindings [2]. Each domain of *DYNC1H1* exhibits a genotype–phenotype correlation in *DYNC1H1*-related disorders [1], [6]. NMD-associated *DYNC1H1* variants are located in the stem domain, whereas those associated with brain involvement and neurodevelopmental disorders are located in the motor or stalk/MTBD domain [5], [6]. Mutations in the stalk domain, which is formed by two MTBD-linked coiled-coil domains [1], reduce the frequency, distance, and speed of the movement of the dynein complex, thus weakening the interaction between the MTBD and microtubules [2] and subsequently interfering with neurodevelopment.

**Fig. 2:**
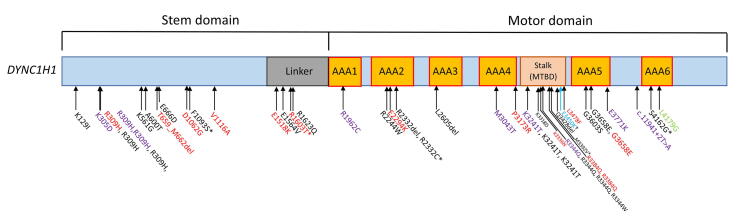
Structure of *DYNC1H1* and distribution of its genetic variants across patients with epilepsy and those with other phenotypes. The arrows indicate the pathogenic variants reported in earlier studies. Green text indicates epilepsy without any malformations of cortical development (MCDs). Red text indicates epilepsy with documented muscle weakness presented as paraparesis or quadriparesis. Purple text indicates undocumented weakness severity but gait/posture disturbance, which indicates muscle weakness or other neurologic deficits. Blue texts with a dagger indicates the case of our patient with a mutation at E3450K. The asterisk indicates the unavailability of MRI data for MCD evaluation. (For interpretation of the references to colour in this figure legend, the reader is referred to the web version of this article.)

The incidence of epilepsy is rare in patients with pathologic *DYNC1H1* variants. By reviewing a total of 20 studies (Table 1), we summarized the characteristics of 48 patients with epilepsy who harbored pathogenic *DYNC1H1* variants [3], [4], [5], [6], [7], [8], [9], [10], [11], [12], [13], [14], [15], [16], [17], [18], [19], [20], [21], [22]. Several clinical, neuroimaging, and genetic characteristics were analyzed (Table 2). Among these patients, the seizure onset was focal, generalized, and unknown, or mixed and early-onset epilepsy was common. The MRI (if available) findings of almost all patients revealed unique cortical malformations, such as pachygyria, hypoplasia/dysplasia corpus callosum, polymicrogyria, heterotopias, and brainstem/cerebellar abnormalities. Furthermore, nearly all patients (95.7 %) had development delay or ID. When present, ID was likely to be severe. Muscle weakness with lower limb predominance was observed in a total of 13 patients. Other characteristic phenotypes were as follows: dysmorphic features (incidence rate, 31.3 %); eye abnormalities (30.2 %), particularly cataract (20.9 %); orthopedic abnormality (26.8 %), particularly pes equinus (17.1 %); and autism (8.3 %). Fig. 2 illustrates the structure of *DYNC1H1* and the distribution of its genetic variant across the associated phenotypes. Exome sequencing data suggest that most genetic variants originate *de novo* and occur in the motor domain or the stalk/MTBD domain (62.5 %). The variants associated with the phenotype of muscle weakness in patients with epilepsy are distributed either in the stem or motor domain.

**Table 1:** Studies reporting epilepsy in patients carrying pathogenic *DYNC1H1* variants and the clinical, genetic, and neuroimaging characteristics of the patients.

Cases	Mutation protein(domain)	Inheritance	Age at epilepsy onset	Seizure type	Neurodevelopment		Cranial MRI/CT	Muscle strength (gait)	Orthopedic abnormality	Eye abnormality	Other malformations
					DD	ID					
Poirier et al., 2013 [4]	p.K129I (stem)	*De novo*	Late onset	ND	Motor delay	Severe ID	Pachygyria (P) and thick CC	NL	NL	NL	NL
Di Dinato et al., 2018 [7]	p.K305D (stem)	*De novo*	1 years	ND	SI	Mild ID	Pachygyria (P > A gradient)	(Unstable gait)	NL	NL	NL
Di Dinato et al., 2018 [7]	p.R309H (stem)	*De novo*	5 months	ND	Global developmental delay	Severe ID	Pachygyria (P > A gradient)	(Sit and stand supported)	NL	NL	Microcephaly
Di Dinato et al., 2018 [7]	p.R309H (stem)	NA	3 months	ND	Motor delay	Severe ID	Pachygyria/agyria (P > A gradient), ventriculomegaly, and cerebellar hypoplasia (vermis and hemisphere)	NA	NL	NL	NL
Di Dinato et al., 2018 [7]	p.R309H (stem)	*De novo*	3 months	ND	Global developmental delay	NL	Pachygyria (P > A gradient)	(Inability to sit)	NL	NL	NL
Minardi et al., 2020 [8]	p.R309H (stem)	*De novo*	1.5 years	Focal-onset, GTCS, and generalized-onset nonmotor	NL	Severe ID	Pachygiria	Tetraparesis	Scoliosis	NL	Cryptorchidism
Accogli et al., 2020 [9]	p.R309H (stem)	NA	NA	ND	Global developmental delay	Severe ID	Pachygyria (P–A gradient), bilateral temporo-occipital subcortical nodular heterotopias, CC hyperplasia, brainstem malformation, and enlarged interthalamic mass	Focal neurological deficits	NL	NL	NL
Jamuar et al., 2014 [10]	p.E561G (stem)	*De novo*	5 years	ND	Motor delay	ID	Pachygyria (P > A gradient) and CC hypoplasia	NA	NA	NA	NA
Accogli et al., 2020 [9]	p.A600T (stem)	NA	NA	ND	Global developmental delay	Severe ID	Pachygyria (P–A gradient) and cavum vergae	Focal neurological deficits	NL	NL	NL
Poirier et al., 2013 [4]	p. T659_M662del (stem)	*De novo*	Early onset	ND	Motor delay	NL	Pachygyria (P) and thin CC	Spastic tetraplegia (bedridden)	NL	NL	Microcephaly
Becker et al., 2020 [6]	p.E666D (stem)	*De novo*	<4 years^1^	Focal onset	Global developmental delay	NL	Pachygyria (P), heterotopia, and pons hypoplasia	NL	NL	Amblyopia	Accessory spleen
Strickland et al., 2016 [3]	p.D1062G (stem)	*De novo*	17 years	Focal to bilateral tonic-clonic	NL	ID	Polymicrogyria (perisylvian region) and CC hypoplasia	Spastic tetraparesis with LL predominance (walker)	NL	Bilateral cataract	NL
Helbig et al., 2016 [11]	p.F1093S (stem)	*De novo*	Infantile	Unknown-onset epileptic spasms	NL	ID	NA	NA	NA	NA	NA
Amabile et al., 2020 [5]	p.V1116A (stem)	*De novo*	4 years	Unknown onset	Global developmental delay	NL	Ventriculomegaly and frontal, temporal, and parietal opercular cortex polymicrogyria	LE and truncal weakness; EMG revealed pure motor neurogenic process	PE	NL	Craniofacial dysmorphism (dolichocephaly), and pectus excavatum
Willemsen et al., 2012 [12]	p.E1518K, (stem)	*De novo*	3 years	Generalized onset	Motor delay and severe SI	NL	Ventriculomegaly and pachygyria (particularly frontal lobes)	Spastic tetraplegia (unwalkable and wheelchair dependent)	PE	Secondary cataract	Craniofacial dysmorphism
Matsumoto et al., 2021 [13]	p.E1564V (stem)	*De novo*	2 months	Focal-onset myoclonic or atonic, FIAS, and generalized-onset tonic	Global developmental delay	Severe ID	Pachygyria (P > A gradient)	Hypotonia	NL	NL	NL
Scoto et al., 2015 [14]	p.R1603T (stem)	*De novo*	NA	ND	Motor delay	NL	NA	LL weakness (crawling and sitting with support)	PE, leg contracture	NL	NA
Di Dinato et al., 2018 [7]	p.R1623Q (stem)	*De novo*	4 months	ND	Global developmental delay	NL	Pachygyria (A > P gradient)	NA	NL	Congenital cataract	NL
Poirier et al., 2013 [4]	p.R1962C (motor)	*De novo*	2 months	Focal onset	Motor delay	Severe ID	Pachygyria (P)	(Awkward gait)	NL	NL	NL
Epi4K Consortium, 2021 [15]	p.R2244W (motor)	*De novo*	13 months	Focal-onset epileptic spasms	SI	NL	Bilateral perisylvian polymicrogyria	NA	Scoliosis	NA	Microcephaly
Becker et al., 2020 [6]	p.E2294K (motor)	*De novo*	<4 years^2^	Focal onset	Motor delay	Severe ID	Pachygyria (A > P gradient, perisylvian) with thick cortex	Severe PLE weakness (broad-based waddling)	PE	NL	NA
Gelineau-Morel et al., 2016 [16]	p.R2332del (motor)	*De novo*	10 years	Generalize onset	Global developmental delay	NL	Bifrontal polymicrogyria	Hypotonia	NA	Bilatera; congenital cataracts, strabismus	Oral dysphagia, gut dysmotility
Tumienė et al., 2018 [17]	p.R2332C (motor)	*De novo*	NA	Focal onset	Global developmental delay	NL	NA	NA	NA	NA	Microcephaly, Autism
Di Dinato et al., 2018 [7]	p.L2605del (motor)	*De novo*	9 months	ND	Global developmental delay	Severe ID	Pachygyria (A > P gradient)	NA	NL	NL	Microcephaly
Kolbjer et al., 2021 [18]	p.M3043T (motor)	*De novo*	11 months	Focal-onset, GTCS, and generalized-onset nonmotor	NL	Severe ID	Pachygyria (temporal > P gradient)	(Spasticity, walk with assistance)	NL	NL	Autism
Becker et al., 2020 [6]	p.P3173R (motor)	*De novo*	<4 years^a^	Focal onset	Global developmental delay	NL	Bifrontal polymicrogyria, periventricular white matter abnormalities, and ventriculomegaly	Severe LL weakness (broad-based waddling with support); NCS revealed sensorimotor neuropathy with axonal loss	NL	Congenital cataract	NA
Poirier et al., 2013 [4]	p.K3241T (stalk/ MTBD)	Familial	2 years 5 months	Focal onset	NL	NL	Pachygyria (P)	NL	NL	NL	NL
Poirier et al., 2013 [4]	p.K3241T (stalk/ MTBD)	Familial	1 year 5 months	Focal onset	Motor delay	Mild ID	Pachygyria (P)	(Awkward gait)	NL	NL	NL
Poirier et al., 2013 [4]	p.K3241T (stalk/ MTBD)	Familial	10 years	Focal onset	NL	NL	Pachygyria (P)	NL	NL	NL	NL
Di Dinato et al., 2018 [7]	p.K3318D (stalk/ MTBD)	NA	6 weeks	ND	Global developmental delay	Severe ID	Pachygyria (P > A gradient), large basal ganglion dysplasia, and tectal hyperplasia	NA	NL	NL	NL
Poirier et al., 2013 [4]	p.K3336N (stalk/ MTBD)	*De novo*	Early onset	ND	Motor delay	NL	Pachygyria (P), frontal polymicrogyria, nodular heterotopia, CC hypoplasia, and brainstem and cerebellum vermis hypoplasia	Spastic tetraplegia (bedridden)	Foot deformities	NL	Microcephaly
Poirier et al., 2013 [4]	p.R3344Q (stalk/ MTBD)	*De novo*	NA	Unknown onset (Lennox-Gastaut syndrome)	Motor delay	Severe ID	Agyria (P), nodular heterotopia, and CC hypoplasia	NA	NL	NL	NL
Poirier et al., 2013 [4]	p.R3344Q (stalk/ MTBD)	*De novo*	5 months	Focal onset	Motor delay	Moderate ID	Pachygyria (P)	(Awkward gait)	NL	NL	Microcephaly
Di Dinato et al., 2018 [7]	p.R3344Q (stalk/ MTBD)	NA	3 months	ND	SI	Severe ID	Dysgyria (P > A gradient, perisylvian), cerebellar hypoplasia (vermis and hemisphere)	NA	NL	NL	NL
Di Dinato et al., 2018 [7]	p.R3344W (stalk/ MTBD)	*De novo*	7 months	ND	Global developmental delay	Severe ID	Pachygyria (P > A gradient)	NA	NL	NL	Sacral dimple
Poirier et al., 2013 [4]	p.R3384Q (stalk/ MTBD)	*De novo*	Early onset	ND	Motor delay	NL	Pachygyria (P), Frontal polymicrogyria, CC dysmorphia, and brainstem and cerebellum hypoplasia	Spastic tetraplegia (bedridden)	Foot deformities	NL	Microcephaly
Chen et al., 2017 [19]	p.R3384Q (stalk/MTBD)	*De novo*	NA	ND	Global developmental delay	NL	Pachygyria (P > A gradient)	Tetraparesis with LL more severe (inability to sit unsupported)	NL	Visual abnormality	Craniofacial dysmorphism (flat posterior cranium, and slightly lower ears)
Lin et al., 2017 [20]	p.M3392V (stalk/ MTBD)	*De novo*	NA	Unknown-onset epileptic spasms	NL	ID	NA	NA	NA	NA	NA
Accogli et al., 2020 [9]	p.Q3428del (stalk/ MTBD)	NA	NA	ND	Global developmental delay	Moderate ID	Bilateral polymicrogyria	NL	NL	NL	NL
Our study	p.E3450K (stalk/MTBD)	Suspected *De novo*	11 months	Focal onset	Global developmental delay	ID	Thin CC and several nodular heterotopias (left centrum semiovale)	NL	NL	Secondary cataract	Craniofacial dysmorphism
Becker et al., 2020 [6]	p.L3478F (stalk/ MTBD)	De novo	<4 years^3^	Generalize-onset atonic	Motor delay	Severe ID	Right ventriculomegaly and white matter hypomyelination	Severe PLE weakness (broad-based waddling); NCS revealed sensorimotor neuropathy with axonal loss	PE	Strabismus	Craniofacial dysmorphism (macrocephaly)
Di Dinato et al., 2018 [7]	p.G3630S (motor)	NA	Approximately ∼ 1 year	ND	Global developmental delay	NL	Pachygyria (P > A gradient), cerebellar hypoplasia (vermis and hemisphere)	NA	PE, bilateral hip dislocation	Congenital cataract	NL
Hertecant et al., 2016 [21]	p.G3658E (motor domain)	*De novo*	6 months	Generalize-onset tonic	Global developmental delay	ID	Pachygyria, bilateral frontoparietal extensive heterotopia, CC hypoplasia, and ventriculomegaly	Hypotonia and drop head	PE	Bilateral congenital cataracts	Microcephaly
Amabile et al., 2020 [5]	p.G3658E (motor)	*De novo*	Early onset	Unknown onset (multifocal epileptiform on EEG)	Global developmental delay	ID	Ventriculomegaly, white matter loss, prominent extra-axial space, and CC hypoplasia	LE weakness (supported step); diagnosed as spinal muscular atrophy	NA	Bilateral cataracts	Ambiguous genitalia
Di Dinato et al., 2018 [7]	pE3771K (motor)	*De novo*	8 months	ND	Global developmental delay	NL	Pachygyria (P > A gradient)	(Inability to sit or stand)	NL	NL	NL
Di Dinato et al., 2018 [7]	c.11941 + 2 T > A	NA	3 years	Focal-onset myoclonic	Global developmental delay	Moderate ID	Dysgyria (P > A gradient)	(Unsteady awkward gait)	NL	NL	Mild dysmorphic features
Johannesen et al., 2019 [22]	p.S4162G (motor)	De novo	13 years	Generalized-onset tonic or nonmotor	NA	NA	NA	NA	NA	NA	NA
Matsumoto et al 2021., [13]	p.L4179S (motor)	De novo	7 years	Focal-onset myoclonic, FIAS, and GTCS	Global developmental delay	Severe ID	NL	NL with slight hypotonia	NL	NL	Autism

Abbreviations: A, anterior; CC, corpus callosum; CT, computed tomography; DD, development delay; EEG, electroencephalography; EMG, electromyography; FIAS, focal impaired awareness seizure; GTCS, generalized tonic-clinic seizure; ID, intellectual disability; LL, lower limb; MRI, magnetic resonance imaging; MTBD, microtubule-binding domain; NA, not applicable; NCS, nerve conduction study; ND, not described; NL, normal; P, Posterior; PE, Pes equinovarus; PLE, proximal lower extremity; SI, speech impairment.

1 In four cases reported in Becker et al., early-onset epilepsy was diagnosed at a median age of 27 (range, 11–48) months.

2 In four cases reported in Becker et al., early-onset epilepsy was diagnosed at a median age of 27 (range, 11–48) months.

3 In four cases reported in Becker et al., early-onset epilepsy was diagnosed at a median age of 27 (range, 11–48) months.

**Table 2:** Clinical and neuroimaging characteristics of patients in *DYNC1H1* variant-associated epilepsy.

Clinical characteristics			Number of patients (N = 48), n (%)
Protein domain of mutation	Stem domain		18 (37.5)
	Motor or stalk domain		30 (62.5)
Inheritance	De novo		**37 (92.5)**
	Familial		3 (7.5)
Epilepsy onset (age)	Early onset (<5 years)		**32 (82)**
	Late onset (≥5 years)		7 (18)
Seizure type	Focal onset		13 (27.1)
	Generalized onset		5 (10.4)
	Unknown onset including epilepsy syndromes (epileptic spasm, Lennox–Gastaut syndrome, and epileptic encephalopathy)		5 (10.4)
	Mixed focal and generalized onset		4 (8.3)
	Not described		21 (43.8)
Neuro-developmental delay	Normal		2 (4.3)
	DD or ID		**45 (95.7)**
	DD		40 (85.1)
	ID		28 (59.6)
	ID severity	Mild	2 (4.3)
		Moderate	3 (6.4)
		Severe	17 (36.2)
		Unknown	6 (12.8)
Magnetic resonance imaging features	Normal		1 (2.3)
	Abnormal		**42 (97.7)**
		Pachygyria	29 (67.4)
		Polymicrogyria	8 (18.6)
		Dysgyria	2 (4.7)
		Ventriculomegaly	4 (9.3)
		Hypoplasia/ corpus callosum dysplasia	11 (25.6)
		Brainstem/cerebellum abnormality	7 (16.3)
		White matter hypomyelination/abnormality	4 (9.3)
		Subcortical nodular heterotopia	6 (14)
Muscle weakness	Normal or without apparent motor involvement		7 (24.1)
	Documented muscle weakness as paraparesis or quadriparesis		13 (44.8)
	Undocumented mueslce weakness severity but gait/posture disturbance		9 (31)
Orthopedic abnormalities	Normal		30 (73.2)
	Abnormality		11 (26.8)
		Pes equinovarus	7 (17.1)
		Scoliosis	2 (4.9)
Ophthalmic abnormalities	Normal		30 (69.8)
	Cataract		9 (20.9)
	Other ophthalmic abnormalities		4 (9.3)
Additional features	Dysmorphic features		15 (31.3)
		Craniofacial dysmorphism	5 (10.4)
		Microcephaly	9 (18.8)
	Autism		4 (8.3)
	Other abnormalities (e.g., genitourinary or gastrointestinal)		6 (12.5)

Abbreviations: DD, developmental delay; ID, intellectual disability.

The two possible pathophysiologies of epilepsy patients with *DYNC1H1* variants are as follows. First, most mutations occur in the motor and stalk/MTBD domains of *DYNC1H1*, affecting brain development; these mutations result in abnormal brain morphologies, including posterior predominant pachygyria, dysgyria, polymicrogyria, microcephaly, nodular heterotopia, and corpus callosum hypoplasia [4], [7], most of which are associated with epilepsy or may serve as epileptic foci. Second, *DYNC1H1* variants (without MCDs) may lead to epilepsy [13]. In an earlier study, *DYNC1H1* was coexpressed with 46 associated epilepsy genes; 15 genes were considered high-confidence genes in the EpilepsyGene database [20]. The findings indicate that *DYNC1H1* is a potential factor associated with epileptic encephalopathies. Together, the aforementioned observations emphasize the importance of *DYNC1H1* in brain development and its epileptogenicity. The other characteristics, including facial dysmorphism and ophthalmic abnormalities, were similar across the patients. Thus, mutations in the motor domain affect not only the brain but also the lens and facial development, resulting in cataract and facial dysmorphism.

Notably, the cases reviewed in the present study reported muscle weakness with lower limb predominance; some patients with this condition presented with axonal neuropathy or motor neuronopathy (assessed through electrophysiological examinations), which may be a manifestation of CMT or SMALED—which are well-known phenotypes of *DYNC1H1* variants. In these cases, *DYNC1H1* variants may not always be located in the stem domain. The genetic variants of MCDs or epilepsy phenotypes are not confined to the motor domain. These findings highlight the overlapping of the clinical phenotypes of various *DYNC1H1*-related disorders.

## 4 Conclusions

We report the case of a patient with a pathogenic *DYNC1H1* variant who presented with rare drug-resistant focal epilepsy. In addition, we summarized the characteristics of patients with *DYNC1H1* variant–related epilepsy by reviewing the relevant literature. To the best of our knowledge, this is the most comprehensive study to explore the association between epilepsy and *DYNC1H1* variants to date. This report expands the phenotypic spectrum of *DYNC1H1*-related disorders to early-onset epilepsy, which is frequently associated with MCDs, neurodevelopmental delay, and multi-systemic involvement. Genetically, they have common mutation sites in the motor domain and mostly occur through *de novo* mutations. Overall, *DYNC1H1* is a key factor for patients wtih developmental and epileptic encephalopathies.

## Consent for publication

The written informed consent was obtained from the patient’s relatives for scientific publication of the case presentation.

## Funding

The study was supported by the Ministry of Science and Technology (MOST 107-2314-B-002-070), Ministry of Science and Technology (MOST 109-2634-F-002-029) and National Taiwan University Hospital (NTUH 109-N4657 and 110-A158). The funder had no role in the design, analyses, collection or interpretation of the data or the decision to submit for publication.

## Authors contributions

CTC drafted the manuscript for intellectual content. TJ and NCL designed of the study and revised the manuscript for intellectual content. CTC, TJ, NCL and CHC was responsible for acquisition of the data. CTC, TJ, NCL, SPF, MZH and YHL was responsible for analysis and/or interpretation of data.

## Ethics approval and consent to participate

Written informed consent was obtained from patient’s relatives for the inclusion of deidentified clinical data in a scientific publication, in accordance with the Declaration of Helsinki.

The study was approved by the Research Ethics Committee of National Taiwan University Hospital (NTUH-REC). The committee’s reference number: (NTUH-REC No: 201505135RINA) and (NTUH-REC No: 201902064RINC).

## Declaration of Competing Interest

The authors declare that they have no known competing financial interests or personal relationships that could have appeared to influence the work reported in this paper.
